# Fetal electrocardiograms, direct and abdominal with reference heartbeat annotations

**DOI:** 10.1038/s41597-020-0538-z

**Published:** 2020-06-25

**Authors:** Adam Matonia, Janusz Jezewski, Tomasz Kupka, Michał Jezewski, Krzysztof Horoba, Janusz Wrobel, Robert Czabanski, Radana Kahankowa

**Affiliations:** 1 Łukasiewicz Research Network – Institute of Medical Technology and Equipment, 118 Roosevelt Str., 41-800 Zabrze, Poland; 2 Silesian University of Technology, Department of Cybernetics, Nanotechnology and Data Processing, 16 Akademicka Str., 44-100 Gliwice, Poland; 3 VSB-Technical University of Ostrava, Department of Cybernetics and Biomedical Engineering, Faculty of Electrical Engineering and Computer Science, 17. Listopadu 2172/15 Str., 70800 Ostrava, Czech Republic

**Keywords:** Biomedical engineering, Data publication and archiving

## Abstract

Monitoring fetal heart rate (FHR) variability plays a fundamental role in fetal state assessment. Reliable FHR signal can be obtained from an invasive direct fetal electrocardiogram (FECG), but this is limited to labour. Alternative abdominal (indirect) FECG signals can be recorded during pregnancy and labour. Quality, however, is much lower and the maternal heart and uterine contractions provide sources of interference. Here, we present ten twenty-minute pregnancy signals and 12 five-minute labour signals. Abdominal FECG and reference direct FECG were recorded simultaneously during labour. Reference pregnancy signal data came from an automated detector and were corrected by clinical experts. The resulting dataset exhibits a large variety of interferences and clinically significant FHR patterns. We thus provide the scientific community with access to bioelectrical fetal heart activity signals that may enable the development of new methods for FECG signals analysis, and may ultimately advance the use and accuracy of abdominal electrocardiography methods.

## Abstract (summary)

**Table:** 

Measurement(s)	heart electrical impulse generation trait • heart function measurement
Technology Type(s)	Internal Fetal Heart Rate Monitoring • electrocardiography
Factor Type(s)	time series • type of fetal electrocardiogram (direct versus abdominal)
Sample Characteristic - Organism	Homo sapiens

Machine-accessible metadata file describing the reported data: 10.6084/m9.figshare.12280007

## Background & Summary

**Fig. 1:**
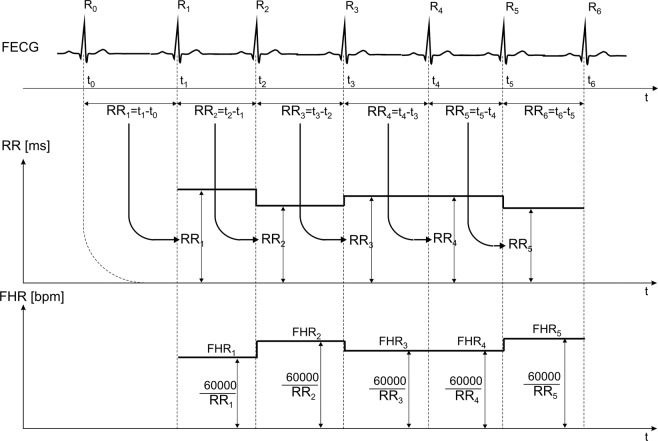
Precise determination of subsequent fetal heart cycle durations (RR_i_) and instantaneous heart rate values (FHR_i_) from the fetal electrocardiogram (FECG), based on the detection of R-waves.

Cardiotocographic monitoring plays an essential role in the assessment of the fetus as it analyses fetal heart rate (FHR) changes against the background of uterine activity and fetal movements^1–3^. Instantaneous FHR values are expressed in beats per minute; they stem from periods between consecutive heartbeats (Fig. 1). The FHR signal is most frequently obtained with the use of the Doppler ultrasound method which serves to monitor mechanical heart activity^4^. If correlation techniques are applied to analyze the periodicity of the ultrasound wave, signal loss is reduced. As a result, FHR analysis is feasible both visually and with the use of computer-aided fetal monitoring systems^2,5^. However, since instantaneous FHR values are averaged, automated beat-to-beat variability evaluation^6^ becomes less reliable, and so do the decisions regarding clinical interventions^7,8^.

For several years now, there has been an increasing interest in monitoring bioelectric activity of the fetal heart^9,10^ because an analysis of the fetal electrocardiogram (FECG) makes it possible to determine FHR with high efficiency and accuracy (Fig. 1). The FECG signal can be recorded invasively – directly from the fetal head during labour^11^, and non-invasively – indirectly from the electrodes placed on the maternal abdominal wall both during pregnancy and labour^12,13^. In the abdominal signals, the amplitude of the fetal QRS complex (FQRS) hardly ever exceeds 20 μV and strongly depends on the maternal body mass index^14,15^. Apart from numerous muscles interferences^16,17^, the strongest one is the maternal electrocardiogram (MECG) of a relatively large amplitude^18–20^. Owing to incomplete MECG suppression^21–24^, the FECG signal becomes unsuitable for further analysis, thus preventing the detection of FQRS complexes, precise location of R waves and FHR determination^25–27^.

The main difficulty in the performance evaluation of various methods to analyze abdominal signals lies in a very limited number of publicly available testing signals with reference information. Our dedicated measurement instrumentation for recording bioelectrical fetal heart activity – the KOMPOREL System^28^ – was deployed to gather pregnancy (antenatal) signals (B1 dataset) and labour (intrapartum) signals (B2 dataset). All signals were recorded in clinical conditions as part of official research projects. The research material presented in this paper contains 10 pregnancy signals (20 minutes each) and 12 labour signals (5 minutes each). Labour signals comprise the abdominal signals (indirect FECG) and the direct reference FECG, simultaneously recorded from the fetal head. For pregnancy signals, an automated multi-stage procedure to detect FQRS complexes was developed in order to provide reference data. The determined locations of all fetal heartbeats (R waves) were validated by clinical experts. For labour signals, the reference FHR signal was obtained on automatically detected FQRS complexes in the direct FECG. It is considered to be the gold standard in terms of the accuracy to determine intervals between subsequent heartbeats^29^. To assess the content and impact of various interferences in abdominal signals, dedicated measures are proposed to quantify amplitude relationships of maternal and fetal signals in relation to interferences. On the other hand, in order to assess the diversity of the material from the point of view of significant FHR variability patterns^30,31^ (e.g. accelerations or decelerations), an automated analysis of the resulting FHR signals was carried out according to the standards applicable in modern obstetrics^2,3^.

Some of the collected signals have already been included in previous studies. The studies focused on developing the methods for MECG suppression in the abdominal signals^32–35^ and on the algorithms to detect FQRS complexes^36–38^, which makes it possible to determine instantaneous FHR with an accuracy close to the direct FECG registration method. The resulting original MECG suppression method relies on precise centering and subtraction of the maternal signal pattern covering a full heart cycle. The algorithm to detect FQRS complexes is based on the detection function using standardized matched filtering and normalization, which significantly reduces sensitivity to muscle disturbances. FQRS complexes, even of an amplitude lower than that of interferences, are correctly detected owing to decision rules in which the cost function is minimized. Since those investigations used only a small part of the signals, the related papers do not describe all the details of the currently presented datasets.

We provide open access to the bioelectrical fetal heart activity signal datasets recorded both during pregnancy and labour, with reference heartbeats annotations. As a result, other researchers may be able to develop more effective methods to extract and analyze the fetal electrocardiogram. We hope that the effectiveness of non-invasive methods for fetal distress assessment will be brought to a level so far achievable by invasive methods only.

## Methods

### Data collection

The developed instrumentation for recording signals was used to collect research material, including four-channel records of bioelectrical fetal heart activity obtained from electrodes placed on the maternal abdominal wall. Pregnancy signals were recorded between the 32nd and 42nd week of pregnancy. Labour signals were obtained in an advanced stage of labour. They consisted of four-channel records of bioelectrical fetal heart activity from abdominal electrodes. Additionally, a direct FECG signal was recorded simultaneously from the fetal head. Labour was monitored between the 38th and 42nd week of gestation, and all signals were collected in clinical conditions as part of research projects at the Department of Obstetrics and Gynecology of the Medical University of Silesia in Katowice, Poland. The research was approved by the competent University Bioethics Committee (Commission approval number NN-013-345/02), and by each of the hospitalized patients.

### Recording protocol

The developed KOMPOREL System for monitoring the bioelectrical activity of a fetal heart was used for recordings. The system consists of a signal recorder module and a portable computer. The recorder module enables simultaneous acquisition of four signals from the maternal abdominal wall as well as one FECG signal directly from the fetal head (during advanced labour only). The recorder exhibits a low level of inherent noise (below 1 µV), a high value of common interference suppression factor (CMRR = 115 dB) and provides signal amplification from the level of several dozen microvolts to several volts^28^. The amplifiers and filters ensure that the FECG band is obtained in the range from 0.05 to 150 Hz. Optionally, power line interferences can be additionally suppressed. If strong low-frequency noise occurs, the cut-off frequency of the high-pass filter increases to 1 Hz. Moreover, the recorder module makes it possible to check the loss of electrode contact, mark fetal movements perceived by the mother, as well as control the charge level of the batteries. The recorded analog signals are digitized with a 16-bit resolution at 500 Hz sampling frequency for abdominal signals and 1 kHz for a direct FECG. The recorded data are archived, a preliminary analysis is performed and signals are visualized by a computer. Recordings along with all additional information are stored in files in the appropriate binary, text or graphic format.

**Fig. 2:**
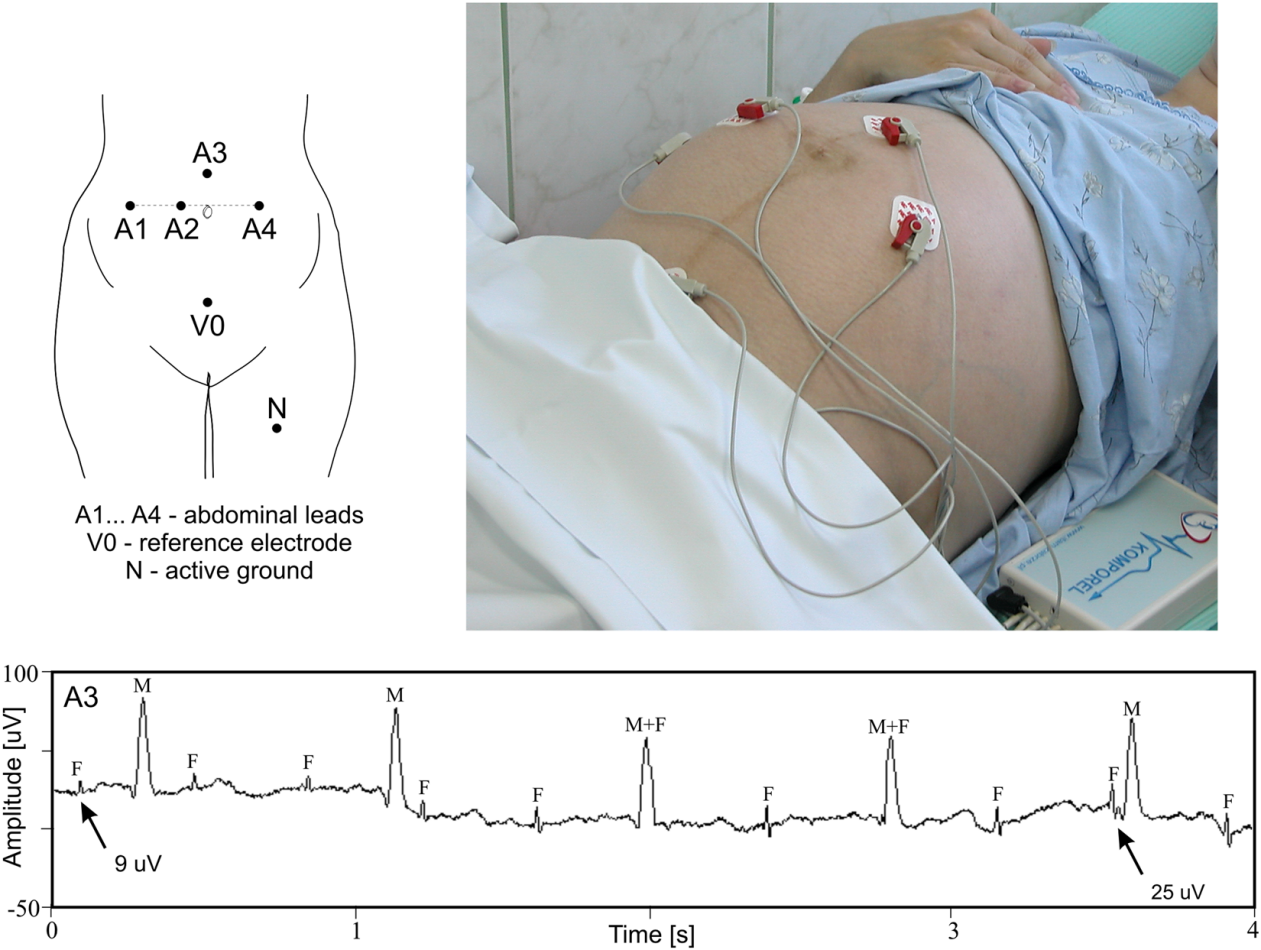
The proposed electrode configuration for the indirect FECG signal recording and an example of a signal recorded on the maternal abdomen (M, F – QRS complexes, maternal and fetal, respectively).

The electrode configuration consists of four measuring electrodes A1 ÷ A4, evenly placed around the patient’s navel line. As a result, signals are recorded relative to one common reference electrode V0 located above the pubic symphysis. Additionally, a common mode reference electrode N (with active-ground signal) is placed on the patient’s left leg. The proposed number and arrangement of electrodes (Fig. 2) result from our investigations and are a compromise between simple application and the development of an effective MECG suppression method to obtain good FECG signal quality. Owing to the appropriate preparation of the abdominal skin (removal of the upper stratum corneum of the epidermis) in the place of application of the measuring electrodes, the level of muscle noise or slow-changing noise was significantly reduced. However, the recorded signals still exhibit low-frequency interferences caused by fetal and/or maternal movements, as well as by impedance changes between the measuring electrodes and maternal skin^16,19,39^.

Standard Ag/AgCl electrodes (3 M Red Dot 2271) were used in the monitoring process, and the top layer of the epidermis was gently wiped with a dedicated conductivity paste (3 M Red Dot Trace Prep 2236). The direct FECG signal was recorded with a sterile spiral electrode (CETRO AB 15133C) placed on the fetal head. All monitoring sessions were carried out by qualified and trained medical staff. Moreover, an experienced biomedical engineer was present (the first author of the presented paper), responsible for observing the recorded signals and selecting the appropriate filter cut-off frequency, as well as controlling the contact of electrodes. Each patient was assigned an identification number, and the gestational age was noted on the monitoring day. While pregnancy signals were being recorded, the patient most often took a position on the left side and a semi-sitting position in labour. The patient could talk freely and change her position if necessary.

### Data processing

All abdominal signals, with sampling frequency of 500 Hz, were initially filtered to suppress power and slow-changing interferences. A simple filter with multiple notches, located every 50 Hz was used^37^:

1 $${\rm{H}}\left({\rm{z}}\right)={{\rm{z}}}^{-50}-\frac{1}{36}{\left(\frac{1-{{\rm{z}}}^{-60}}{1-{{\rm{z}}}^{-10}}\right)}^{2}$$

The first cutoff frequency is equal to approximately 5 Hz, which assures effective suppression of low frequency noise. The top-band between 45 Hz and 55 Hz results in successful elimination of the powerline interference. In the case of a direct FECG signal, the initial noise suppression was carried out in a similar way, with the filter parameters adjusted to the sampling frequency of 1 kHz.

The results of preliminary automated detection of fetal QRS complexes were verified by clinical experts, who also corrected manually individual fetal heartbeat locations. The signals together with the interpretation provided by experts in the form of annotations about the locations of R waves are the main part of the developed datasets. Additionally, based on the annotated R-wave locations, the reference heart rate signals were determined and subsequently analyzed for a detailed quantitative assessment of clinically relevant FHR variability patterns. All pre-filtered signals were evaluated in terms of mutual amplitude relationships of the maternal and fetal signals and their relation to other interferences (mainly muscle) occurring in abdominal signals. The obtained results describe the research material and complement the information on particular abdominal signals. A detailed description of the proposed methodology for signal processing and analysis is presented below, separately for B1 and B2 datasets.

### Pregnancy (antenatal) signals

The first stage of the abdominal signal analysis is the suppression of a dominant MECG signal. An innovative method was proposed, based on subtracting the maternal signal pattern covering the full heart cycle – PQRST complex. The pattern is created by averaging the relevant signal fragments and is constantly updated to keep up with small changes in the MECG shape. What matters most for the correct operation of the method is the precise determination of fiducial points, locating the maternal R waves, as well as the selection of coefficients that scale pattern amplitudes in the subtraction process^34,40^. After MECG suppression, each record in the dataset included four FECG components along with the remaining noise. Quite often, detection of FQRS complexes in a given signal was relatively simple due to their large amplitude, although equally often it was very difficult when the level of muscle interferences was comparable to the amplitude of fetal R waves. Therefore, the best quality FECG signal selected with dedicated measures was used to detect fetal QRS complexes. In order to determine the signal quality index based on the quasi-periodicity of the signal, the autocorrelation function was used, calculated in windows of width being adjusted to the fetal heart rate^41^. Automated pre-detection and manual correction of fetal R-wave positions were applied after selecting the best quality FECG signal. According to^37^, K different detection functions were implemented. Their amplitudes were compared with a continuously modified detection threshold and a simple decision rule. As a result, K numerical sequences {R_k_(i)} were provided to describe the locations of the detected function peaks. The RR_k_ intervals were determined for each k series:

2 $${{\rm{RR}}}_{{\rm{k}}}({\rm{i}})={{\rm{R}}}_{{\rm{k}}}({\rm{i}}+1)-{{\rm{R}}}_{{\rm{k}}}({\rm{i}})$$

where: k = 1, 2,…, K means the k-th number series for a given k-th detection function, i = 1, 2, …, I_k–1_ denotes the i-th RR interval, and I_k_ represents the number of peaks detected using the k-th detection function.

Then, for each RR_k_(i) series, the value of the functional Δ_k_ describing its length variability was calculated:

3 $${\Delta }_{{\rm{k}}}=\frac{{\sum }_{{\rm{i}}=1}^{{{\rm{I}}}_{{\rm{k}}}-3}\left|\left[{{\rm{RR}}}_{{\rm{k}}}({\rm{i}}+2)-{{\rm{RR}}}_{{\rm{k}}}({\rm{i}}+1)\right]-\left[{{\rm{RR}}}_{{\rm{k}}}\left({\rm{i}}+1\right)-{{\rm{RR}}}_{{\rm{k}}}({\rm{i}})\right]\right|}{{{\rm{I}}}_{{\rm{k}}}-3}$$

For the second stage of the analysis, the results of the detection function for which the functional ${{\rm{k}}}_{{\rm{opt}}}$ had a minimum value were selected:

4 $${{\rm{k}}}_{{\rm{opt}}}=\begin{array}{c}{\rm{\arg }}({\rm{\min }}\,{\Delta }_{{\rm{k}}})\\ 1\le {\rm{k}}\le {\rm{K}}\end{array}$$

The second stage involved manual correction of the FQRS locations. For that purpose, clinical experts were presented with a properly scaled FECG signal fragment together with the selected detection function. Additionally, the abdominal signal fragment along with maternal QRS markers were displayed to facilitate the task. Signal shifts in time caused by filtering were removed by appropriate correction delays. The expert’s potential task was to remove an erroneous redundant detection, add a new, previously undetected FQRS complex or correct the position of the fiducial point determining the fetal R-wave. In accordance with this procedure, the fetal R-wave reference positions were determined for each record. Also, places were marked where detection of complexes was unreliable because the level of interferences was too high. For that purpose, an additional detection reliability flag was used, whose value 0 means unreliable detection and value 1 – a correct determination of the FQRS position. It is recommended that imprecisely detected heartbeat points should be omitted, for example when evaluating the detection efficiency. Moreover, it should be noted that the “reference” fetal heart rate signal in pregnancy records is accurate to a limited extent only, which is the result of numerous interferences significantly affecting the accuracy of individual R-wave locations. Nevertheless, the proposed approach is commonly used for analyzing FECG signals recorded during pregnancy if accurate reference data cannot be obtained^27,42–44^.

### Labour (intrapartum) signals

Records from labour monitoring include an indirect fetal electrocardiogram from four abdominal leads and a direct FECG signal recorded simultaneously from a fetal head. Direct FECG signal registration is the most reliable for the accurate determination of instantaneous FHR values^29^. Unfortunately, if recording takes place during active labour, it is extremely difficult to ensure good signal quality throughout the recording session due to frequent loss of contact between the electrode and the fetal head. This is caused by maternal movements or cyclical palpation to assess the progress of labour. On the other hand, it is during labour that the measurement environment is the most adverse when it comes to the quality of the FECG indirectly recorded from the abdominal wall^45^. The evaluation of how effective the detection of FQRS complexes is in these signals and in such conditions provides the most reliable information on the usefulness of abdominal electrocardiography. For intrapartum signals, the FHR reference signal was determined on the basis of the automated detection of R waves in a direct FECG signal and subsequently verified by clinical experts. For the detection of FQRS complexes, an algorithm using normalized matched filtering was applied to reduce sensitivity to interferences. The algorithm was accompanied by decision rules based on minimizing the cost function for predicting the length of consecutive fetal heart cycles. A detailed description of the fetal QRS detection algorithm is provided in^37^. The obtained data are the “gold standard”, which is the precise FHR reference signal. The duration of each correctly verified signal was 5 minutes.

### FHR signal characteristics

While visually interpreting the FHR signal a clinician tries to identify those signal features that indicate fetal well-being (e.g. accelerations being the rising heart rate relative to the FHR baseline that may be a response to fetal movement) and those that may be signs of fetal distress (e.g. decelerations being the lowering heart rate in relation to the FHR baseline that may be a result of uterine contraction). Based on the recognized episodes, the clinician interprets a given record as normal, abnormal or suspicious. This, in turn, implies further actions: prolonged monitoring, an additional ultrasound examination or even a decision on earlier pregnancy termination^46^. An automated signal analysis also includes the detection of acceleration/deceleration episodes, tachycardia/bradycardia periods, as well as the estimation of the instantaneous FHR variability^47,48^ that cannot be seen with the naked eye. The automated interpretation of the monitoring process using a reliable quantitative description of the FHR signal, which effectively assists the clinician in fetal state evaluation, is the main aspect of the clinical usefulness of modern fetal monitoring systems^3,8,30^. The reference pregnancy and labour FHR signals were subjected to the classic automated analysis using the computer-aided fetal monitoring MONAKO System. The analysis aimed to assess the diversity of the FHR signals in time domain given the type and number of recognized FHR variability patterns. The FHR signals represented in a form of time series of events were initially sampled at a frequency of 4 Hz (which is a standard in fetal monitoring based on the Doppler ultrasound method). The results of signal analysis were available both in numerical form and as graphic markers presented directly on FHR traces, e.g. duration and maxima/minima of the detected acceleration/deceleration patterns, respectively.

### Amplitude relationships in abdominal signals

To analyze amplitude relationships of individual components in abdominal signals, dedicated measures based on Signal-to-Noise Ratio (SNR) were proposed, and the sum of low frequency, power line and muscle interferences was assumed as noise^33^. Muscle interferences are generated mainly by abdominal muscles, and they also result from the contraction activity of the uterus. Low-frequency and power line interferences are basically suppressed during preliminary signal filtering. However, muscle interferences are much more difficult to suppress because in a wide frequency range they coincide with the FECG signal. Based on the assumption that the amplitude of P and T waves is relatively small, a mean power of interferences PN was determined in the abdominal signal as a mean signal power outside the maternal and fetal QRS complexes. Further on, based on the assumption that there is no correlation between the useful signal and interferences, a mean MECG signal power PM was calculated as the difference between the signal power in places where only maternal QRS complexes (MQRS) occur (in the absence of coincidence with FQRS complexes) and the power of interferences. The mean FECG signal power PF was determined in an analogous way. The diversity of abdominal signals given the characteristics of particular components was described by two basic indices that allow relationships of amplitudes of MECG and FECG signals to be assessed in relation to interferences:

- Index determining the MECG signal level in relation to interferences:
  5 $${\rm{WM}}=10\cdot {\rm{\log }}\frac{{{\rm{P}}}_{{\rm{M}}}}{{{\rm{P}}}_{{\rm{N}}}}$$
- Index determining the FECG signal level in relation to interferences:

6 $${\rm{WF}}=10\cdot {\rm{\log }}\frac{{{\rm{P}}}_{{\rm{F}}}}{{{\rm{P}}}_{{\rm{N}}}}$$

Based on the difference between the indices, we can directly estimate the value of the WMF index, which indicates how many times the amplitude of the interfering maternal signal is higher than the useful FECG component.

In addition, a new index was proposed to describe energy changes of the MQRS and FQRS complexes in a given abdominal signal because lower QRS energy variation makes it possible to obtain a more uniform detection function and thus to increase the effectiveness of detecting subsequent heartbeats^14,19,49–51^. After determining a mean QRS complex the coefficients of amplitude change for particular complexes, relative to a mean QRS, were determined according to the following formula:

7 $${{\rm{r}}}_{{\rm{i}}}=\frac{{\sum }_{{\rm{k}}=1}^{{\rm{S}}}\left\{{{\rm{QRS}}}_{{\rm{avg}}}({\rm{k}})\right\}\cdot \left\{{{\rm{QRS}}}_{{\rm{i}}}({\rm{k}})\right\}}{{\sum }_{{\rm{k}}=1}^{{\rm{S}}}{{\rm{QRS}}}_{{\rm{avg}}}^{2}({\rm{k}})}$$

where: r_i_ – is the amplitude change factor calculated for the i-th QRS complex, S – is the band width expressed in samples: MQRS = 100 ms, FQRS = 40 ms, QRS_avg_(k) and QRS_i_(k) – denote the k-th sample of the mean complex and the k-th sample of the i-th complex.

On the basis of r_i_ coefficients, calculated separately for MQRS and FQRS, indices characterizing energy changes of MQRS complexes in abdominal signals – WEM, and FQRS complexes – WEF were defined:

8 $${\rm{WEM}}=\frac{\sqrt{\frac{1}{{\rm{J}}-1}\cdot {\sum }_{{\rm{i}}=1}^{{\rm{J}}-1}\left({{\rm{r}}}_{{\rm{i}}+1}-{{\rm{r}}}_{{\rm{i}}}\right)}}{\frac{1}{{\rm{J}}}\cdot {\sum }_{{\rm{i}}=1}^{{\rm{J}}}{{\rm{r}}}_{{\rm{i}}}}\,{\rm{WEF}}=\frac{\sqrt{\frac{1}{{\rm{I}}-1}\cdot {\sum }_{{\rm{i}}=1}^{{\rm{I}}-1}\left({{\rm{r}}}_{{\rm{i}}+1}-{{\rm{r}}}_{{\rm{i}}}\right)}}{\frac{1}{{\rm{I}}}\cdot {\sum }_{{\rm{i}}=1}^{{\rm{I}}}{{\rm{r}}}_{{\rm{i}}}}$$

where: J – is the number of MQRS complexes that do not overlap with FQRS complexes, I – denotes the number of FQRS complexes that do not overlap with MQRS complexes.

Ideally, in the absence of changes in both the amplitude and shape of particular QRS complexes in a given signal the above indices are equal to zero. Index values are also affected by the level of interferences. If the amplitude of interference is comparable to a QRS amplitude, then index values take that into account. Unfortunately, in abdominal signals it is difficult to separate the FECG signal from muscle interferences, hence it is difficult to reliably estimate the energy changes of particular QRS complexes.

## Data Records

The shared data were divided into two subsets marked *B1_Pregnancy_dataset* and *B2_Labour_dataset*. The subsets are stored on a server at the generalist repositories (*figshare*) integrated with Scientific Data^52^.

Until now, no record from the presented B1 pregnancy signal dataset has been made publicly available to scientists. As for the B2 dataset containing labour signals, the five records (r01, r04, r07, r08, r10) constituting only 9.6% of the duration of all currently presented abdominal signals, were used in previous publication^36^, and then made accessible through the *PhysioNet*^53^. In those records, the abdominal signals were resampled at 1 kHz for easier comparison with a direct FECG signal. The currently proposed B2 dataset contains 12 records accompanied by a detailed description of the amplitude relationships of individual components in abdominal signals, as well as the characteristics of clinically significant patterns in the FHR reference signal. Annotations indicating the location of fetal QRS complexes (developed on the basis of a direct FECG analysis) were extended by annotations indicating the location of maternal heartbeats. Each record was assigned numerical and graphic results of an automated analysis of the FHR signals important for fetal assessment.

### B1_Pregnancy_dataset

The B1 pregnancy signal dataset consists of 10 records marked *B1_Pregnancy_X* (where *X* is a record number), each containing four files (duration of signals: 20 minutes, sampling frequency: 500 Hz). In the binary file *B1_abSignals_X.ecg* saved in the LabView format there are four abdominal signals after preliminary filtering and four indirect FECG signals after the suppression of the interfering MECG by subtracting the maternal PQRST complex pattern and the first derivative of the QRS complex. Additionally, all signals were saved in the text file *B1_abSignals_X.txt*. The text file *B1_Maternal_R_X.txt* contains information (expressed as a sample number) on the fiducial points that indicate the locations of maternal QRS complexes (R-waves) in abdominal signals. The file *B1_Fetal_R_X.txt* contains information on the fiducial points for fetal QRS complexes in FECG signals. The fiducial points for FECG have been verified by clinical experts. Hence, each point has an attached reliability flag. If the flag equals 0, it means that the fetal R-wave position could not be verified by an expert due to a high level of interferences. On the other hand, 1 means that the R-wave position was correctly verified. A total of 18,936 MQRS and 28,405 FQRS complexes were found in the pregnancy dataset. The number of unverified FQRS complexes was 130 (0.46%). If the mean duration of the fetal heart cycle in B1 is assumed to be 421.1 ms (60000/142.5 bpm), this results in the FHR signal loss of less than 55 s in the entire 200-minute research material. For the value of 100 ms as the mean duration of the MQRS complex and 40 ms as the duration of FQRS, the number of maternal and fetal complexes for which coincidence did not occur was determined: 8,106 and 17,575 complexes, respectively.

The content of the B1 pregnancy signal dataset was analyzed in terms of the diversity of MECG and FECG amplitude relationships, as well as their relation to other interferences; mainly muscular as the analysis was performed on abdominal signals after suppression of low-frequency and power line interferences. Complete data are available in an online version only (Online-only Table 1), in the main subset of *B1_Pregnancy_dataset*. In this work, the discussed results relate only to mean values for all records. The values were obtained on the basis of the results from four particular abdominal channels (Table 1). It is clear that the amplitude of the dominant MECG in relation to interferences is more than five times higher – the mean value of the WM index is 14.3 dB. The WM value for particular records varies, but it always exceeds 10.9 dB. The mean value of the WF index describing the amplitude relationships of the FQRS to interferences is 3.4 dB. However, there are records for which WF is as high as 5 dB – the FQRS amplitude is almost twice as high as the interferences. For some recordings WF is negative, i.e. the FQRS level is below the interference amplitude. This provides proof for an unfavourable measuring environment, although such cases are very valuable in the process of validation of automated algorithms for the detection of FQRS complexes^18,36^. At the same time, the WMF index, whose mean value in this case is almost 11 dB, shows that the amplitude of MQRS complexes is about 3.5 times higher than FQRS (although the range is relatively wider: from 8.5 to 17.7 dB). It can be inferred from the analysis of the WEM and WEF indices describing changes in the amplitude of MQRS and FQRS complexes that the level of the MQRS amplitude is stable in all records – the WEM values are very close to the mean value of just 0.09. In turn, the amplitude of FQRS complexes in particular records changes much more, as evidenced by the high mean WEF value of 0.52. This is also significantly impacted by muscle interferences, the level of which is comparable to FQRS complexes.

**Table 1:** The mean values of SNR indices describing the diversity of particular records from the B1_Pregnancy_dataset.

Record	WM [dB]	WF [dB]	WEM	WEF
B1_Pregnancy_01	16.5	5.3	0.05	0.45
B1_Pregnancy_02	14.1	4.1	0.08	0.48
B1_Pregnancy_03	15.0	5.0	0.09	0.40
B1_Pregnancy_04	12.5	4.0	0.14	0.51
B1_Pregnancy_05	17.5	8.1	0.06	0.18
B1_Pregnancy_06	12.9	4.4	0.11	0.31
B1_Pregnancy_07	11.6	0.8	0.11	1.06
B1_Pregnancy_08	10.9	−1.6	0.10	0.41
B1_Pregnancy_09	16.9	−0.7	0.05	0.79
B1_Pregnancy_10	14.9	5.1	0.07	0.59
**Mean**	**14.3**	**3.4**	**0.09**	**0.52**

**Fig. 3:**
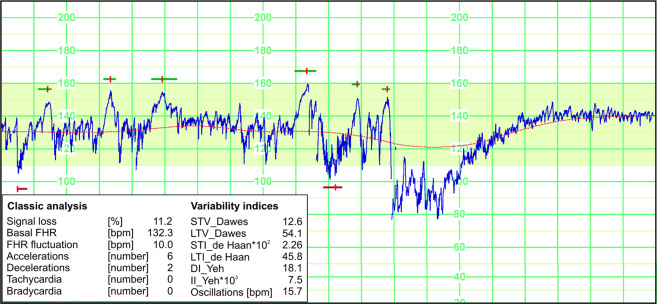
An example of the FHR signal (Record 10 in Table 3) recorded in pregnancy which includes six accelerations and two decelerations; the basal FHR is low – about 132 bpm.

The pregnancy dataset was analyzed for the occurrence of clinically significant patterns in FHR signals by means of a computer-aided fetal monitoring system. The system input data (Fig. 3) were the reference FHR signal, determined on the basis of R-wave positions in abdominal signals (corrected by experts). For each record from the pregnancy dataset, the FHR waveform with a graphic presentation of analysis results was placed in an additional graphic file called: *B1_FHR_analysis_X.jpg*, which is located in a given data record. As can be seen in Table 2, the mean FHR value for all pregnancy records was 142.5 ± 9.0 bpm (a range from 128.3 to 156.3 bpm).

**Table 2:** The results of the FHR analysis for the B1 pregnancy dataset and B2 labour dataset.

FHR variability parameter	B1_Pregnancy_dataset	B2_Labour_dataset
Basal FHR [bpm]	128.3–156.3* 142.5 ± 9.0^#^	125.4–152.4 133.0 ± 7.2
FHR fluctuation [bpm]	7.2–15.4 11.6 ± 2.2	6.4–14.7 8.3 ± 2.3
Accelerations	0–10	0–3
Decelerations	0–2	0–1
Oscillations [bpm]	9.0–17.9 13.0 ± 3.3	8.0–20.9 13.8 ± 4.4
LTV_Dawes [ms]	25.3–58.6 38.2 ± 11.2	28.0–66.8 46.1 ± 13.0
STV_Dawes [ms]	2.9–12.6 6.4 ± 3.2	3.1–10.5 7.0 ± 2.7

*Min – Max. ^#^Mean ± standard deviation.

The mean FHR fluctuation was at the level of 11.6 ± 2.2 bpm (a range from 7.2 to 15.4 bpm). Varying numbers of acceleration and deceleration patterns were detected in the records. There were records without any acceleration and those in which as many as ten acceleration patterns were detected. The number of decelerations ranged from 0 to 2. The mean value of FHR oscillations for pregnancy records was 13.0 ± 3.3 bpm (a range from 9.0 to 17.9 bpm). The B1 dataset exhibited a large range of values of the indices describing instantaneous FHR variability. The mean value of the LTV_Dawes long-term variability index was 38.2 ± 11.2 ms (a range from 25.3 to 58.6 ms), while for STV_Dawes short-term variability it amounted to 6.4 ± 3.2 ms (a range from 2.9 to 12.6 ms). Detailed FHR analysis results for particular pregnancy records are provided in Table 3.

**Table 3:** Results of the FHR analysis for the pregnancy dataset records (*B1_Pregnancy_01* to *B1_Pregnancy_10*) comprising: Signal loss (**Loss**), Basal FHR (**Basal**), FHR fluctuations (**Fluct**), number of accelerations (**Acc**) and decelerations (**Dec**), oscillation range (**Osc**), as well as the indices describing instantaneous FHR variability (**LTV**_Dawes, **STV**_Dawes, **LTI**_de_Haan, **II**_Yeh, **DI**_Yeh).

Record	Loss [%]	Basal [bpm]	Fluct [bpm]	Acc	Dec	Osc [bpm]	LTV [ms]	STV [ms]	LTI	STI	II	DI
01	0.2	156.3	14.1	10	0	17.5	43.3	5.2	25.1	0.0113	0.0076	8.8
02	0.6	140.3	11.2	0	1	17.9	58.6	8.9	28.6	0.0128	0.0087	15.3
03	0.6	128.3	7.2	5	0	10.3	38.8	10.1	31.0	0.0201	0.0050	13.6
04	0.0	139.0	11.7	3	0	10.5	32.1	4.0	17.6	0.0080	0.0053	5.8
05	0.2	138.8	10.5	4	0	10.5	25.3	2.9	11.4	0.0054	0.0037	4.4
06	0.2	144.7	11.3	2	0	9.0	25.8	3.7	12.9	0.0070	0.0042	5.9
07	1.0	155.9	15.4	2	0	13.6	34.2	6.6	14.6	0.0112	0.0057	12.2
08	0.6	145.8	12.5	5	0	14.3	39.8	5.5	21.7	0.0091	0.0065	9.4
09	2.5	143.5	11.6	3	0	10.2	30.0	4.2	17.1	0.0080	0.0049	6.6
10	11.2	132.3	10.0	6	2	15.7	54.1	12.6	45.8	0.0226	0.0075	18.1

### B2_Labour_dataset

The B2 labour signal dataset consists of 12 records marked *B2_Labour_X*, where X is a record number with each record containing five data files. In the binary file *B2_abSignals_X.ecg* saved in the LabView format there are four abdominal signals after preliminary filtering and four FECG signals after the suppression of interfering MECG (signal duration: 5 minutes, sampling frequency: 500 Hz). The binary file *B2_dFECG_X.ecg* saved in the same format contains a direct FECG signal (raw and after preliminary filtering), registered simultaneously from a fetal head (signal duration: 5 minutes, sampling frequency: 1 kHz). All signals are also available in the text files marked as *B2_abSignals_X.txt* and *B2_dFECG_X.txt*, respectively. The text file *B2_Maternal_R_X.txt* contains information (as a sample number) about fiducial points indicating the locations of maternal QRS complexes (R waves) in abdominal signals. The text file *B2_Fetal_R_X.txt* provides information about fiducial points that indicate the locations of fetal QRS complexes in the direct FECG signal.

A total of 7,903 FQRS complexes were detected in all labour signals; the complexes were regarded as a reference. The locations of fetal R waves were described with an accuracy of 1 ms. At the same time, 5,177 MQRS complexes were detected in particular abdominal signals. The number of fetal and maternal QRS complexes without coincidence was 5,142 and 2,415, respectively. As can be seen in Table 4, for all abdominal signals recorded during labour, the dominant component is also the MECG, whose amplitude in relation to interferences is more than three times higher; the mean value of the WM index is 10.4 dB. The WM value for particular records is different, but it always exceeds 6.5 dB. The mean value of the WF index, describing the relationships between the amplitude of FQRS and muscle interferences, is 3.6 dB. However, there are records for which WF is around 7 dB (the FQRS amplitude is over two times higher than interference). A case has also been observed where WF is close to − 2.7 dB, which means that the FQRS level is below the interferences. At the same time, following an analysis of the WMF indicator, whose mean value is 6.8 dB (a range from 1.8 to 10.9 dB), an increase in the amplitude of the FQRS during labour can be noticed, compared to the maternal component. It can be inferred from the analysis of the WEM and WEF indices describing the changes of the MQRS and FQRS complexes amplitude in labour signals that the FQRS amplitude level is more stable compared to pregnancy signals. This is confirmed by a lower mean WEF value of 0.33. On the other hand, WEM demonstrates greater changes than for pregnancy signals, with a mean of 0.2 and range from 0.1 up to 0.36. Strong and irregular interferences from labour related uterine contractions have a significant impact on all determined index values. The obtained results relate to mean values for particular records. The values were calculated on the basis of the results from four abdominal channels. Detailed data are available only in an online version (Online-only Table 2) located in the main subset of *B2_Labour_dataset*.

**Table 4:** The mean values of SNR indices describing diversity of particular records in the B2_Labour_dataset.

Record	WM [dB]	WF [dB]	WEM	WEF
B2_Labour_01	10.6	6.7	0.20	0.20
B2_Labour_02	12.6	5.0	0.10	0.25
B2_Labour_03	7.8	−2.7	0.30	0.54
B2_Labour_04	11.7	2.3	0.13	0.31
B2_Labour_05	9.7	5.9	0.28	0.20
B2_Labour_06	12.8	3.7	0.10	0.19
B2_Labour_07	11.4	0.5	0.27	0.67
B2_Labour_08	10.6	7.0	0.18	0.18
B2_Labour_09	11.4	3.9	0.15	0.44
B2_Labour_10	11.6	0.7	0.11	0.48
B2_Labour_11	8.4	6.6	0.21	0.16
B2_Labour_12	6.5	3.8	0.36	0.28
**Mean**	**10.4**	**3.6**	**0.20**	**0.33**

The labour dataset content was analyzed for occurrence of patterns in FHR signals by analogy to the B1 pregnancy dataset. In this case, the reference FHR signal, determined on the basis of a direct FECG signal analysis and additionally verified by experts, formed the input data for a computer-aided fetal monitoring system. For each record from the B2 dataset, the FHR waveform and a graphic presentation of its analysis results are placed in an additional graphic file called: *B2_FHR_analysis_X.jpg*, included in a given data record.

The mean FHR value for all labour records (Table 2) was 133.0 ± 7.2 bpm (a range from 125.4 to 152.4 bpm). The mean FHR fluctuation was at the level of 8.3 ± 2.3 bpm (a range from 6.4 to 14.7 bpm). In total, 13 acceleration and 1 deceleration patterns were detected. The relatively small number of automatically detected decelerations in B2 results from a relatively short signal duration. A visual inspection of particular FHR records shows that there are cases where the recording started during a deceleration (e.g. *B2_FHR_analysis_02.jpg*) as well as cases ending in an ongoing deceleration (e.g. *B2_FHR_analysis_03.jpg*). Unfortunately, such patterns are not automatically classified as decelerations. The mean value of FHR signal oscillations was 13.8 ± 4.4 bpm (a range from 8.0 to 20.9 bpm). The mean value of the LTV_Dawes long-term variability index amounted to 46.1 ± 13.0 ms (a range from 28.0 to 66.8 ms), while the short-term variability index STV_Dawes was 7.0 ± 2.7 ms (a range from 3.1 to 10.5 ms). Detailed results of the analysis of particular labour records are provided in Table 5.

**Table 5:** The results of the FHR analysis for the labour dataset records (*B2_Labour_01* to *B2_Labour_12*) comprising: Signal loss (**Loss**), Basal FHR (**Basal**), FHR fluctuations (**Fluct**), number of accelerations (**Acc**) and decelerations (**Dec**), oscillation range (**Osc**), as well as the indices describing the instantaneous FHR variability (**LTV**_Dawes, **STV**_Dawes, **LTI**_de_Haan, **II**_Yeh, **DI**_Yeh).

Record	Loss [%]	Basal [bpm]	Fluct [bpm]	Acc	Dec	Osc [bpm]	LTV [ms]	STV [ms]	LTI	STI	II	DI
01	1.7	128.7	7.2	2	0	12.9	44.9	6.1	20.1	0.0081	0.0065	10.3
02	5.8	131.9	7.6	1	0	19.9	66.8	9.5	46.9	0.0163	0.0108	13.9
03	27.5	152.4	14.7	0	0	20.9	52.7	6.8	21.0	0.0101	0.0077	12.9
04	2.5	137.5	9.2	2	0	14.8	46.7	9.3	28.8	0.0182	0.0071	13.3
05	1.7	132.0	6.9	3	0	18.6	66.1	10.5	35.7	0.0122	0.0081	16.9
06	0.0	136.7	9.5	1	0	10.4	33.4	3.1	19.7	0.0046	0.0057	5.0
07	0.0	126.2	6.4	2	1	14.3	51.9	10.0	57.1	0.0169	0.0080	13.7
08	0.0	128.9	7.2	0	0	10.4	36.9	6.1	12.9	0.0092	0.0048	9.0
09	0.8	134.7	9.0	0	0	10.7	36.3	4.0	19.5	0.0070	0.0054	5.8
10	0.0	125.4	6.7	0	0	8.4	33.1	4.6	10.6	0.0075	0.0043	6.3
11	3.3	130.3	7.1	2	0	16.8	56.9	9.5	35.6	0.0141	0.0086	14.9
12	0.0	131.3	8.1	0	0	8.0	28.0	4.3	18.3	0.0076	0.0044	6.1

## Technical Validation

Modern perinatal medicine is expected to enable the mother to give birth to a healthy and well-developed child. It is much more difficult to assess the condition of a fetus than to determine the health status of a child or an adult. The most common cause of perinatal morbidity and mortality is intrauterine hypoxia. The fetus responds to hypoxia with cardiovascular system changes, which results in fetal heart rate (FHR) variability. This indirectly affects oxygenation and functioning of the central nervous system^1,54^. Analysis of FHR variability, together with uterine muscle contractions and fetal movement activity, subject to cardiotocographic monitoring, currently play a fundamental role in fetal assessment^2,55^. The strength of cardiotocography lies in that normal ranges of the results of the FHR signal analysis almost always (>95%) confirm fetal wellbeing. Unfortunately, questionable or abnormal signal features may indicate both fetal distress and its absence^30,31^. The classic fetal heart rate variability analysis consists in determining the FHR baseline and then in identifying bradycardia/tachycardia, acceleration/deceleration patterns as well as the type and amplitude of oscillations. A more advanced FHR analysis is aimed at evaluating instantaneous FHR variability, especially with beat-to-beat approach, which is very important in fetal condition assessment^3,5^. The most common technique of recording the FHR signal is the Doppler ultrasound method, which makes it difficult to correctly estimate the instantaneous heart rate variability because the measurements are averaged. On the other hand, instantaneous heart rate variability plays a crucial role in the diagnosis of fetal condition^7,29^.

The above limitations inspired us to search for other, more effective ways to evaluate fetal condition. The increased interest in monitoring the fetal cardiac bioelectrical activity is due to the fact that the analysis of the fetal electrocardiogram ensures high efficiency and accuracy of the heart rate measurement even on a beat-to-beat level. The first fetal electrocardiogram was recorded as early as in the 1960-ies by means of a special spiral electrode enabling direct signal registration from a fetal head^11^. Due to the way the electrode is placed, its use is limited only to the advanced labour stage. The 1960-ies also saw the development of the first instrumentation that allowed FHR monitoring from the electrocardiogram, as well as of the set of dedicated parameters (used to date) to quantify FHR variability, supporting early recognition of fetal intrauterine hypoxia^7,47^. However, all the descriptive parameters require a highly accurate determination of instantaneous FHR values, and they, in turn, require a signal represented as time series of events^4,29^. Direct FECG registration, due to its invasiveness and application limited only to the advanced labour stage, has not been widely used.

An alternative method is to record the FECG indirectly – from electrodes placed on the surface of the maternal abdomen. This method is non-invasive and can be used both during pregnancy and labour. The method cannot be applied on a wider scale owing to the poor quality of the FECG signal recorded in the presence of numerous interferences^10,19^. Undoubtedly, the evaluation of its usefulness requires, above all, the effectiveness of the FQRS detection to be measured, which directly affects the continuity of FHR signal determination^18,26^. It depends on the entire abdominal signal processing channel: interference filtering, maternal electrocardiogram suppression and FQRS complexes detection^17,39,56^. The effectiveness estimates the usefulness of the FHR signal based on a non-invasive FECG for classic assessment of heart rate variability. In the literature^12,14,18,22,36,37^ several different indicators have been defined to assess the effectiveness of the FQRS complex detection. The most objective ones are as follows: Performance Index (PI), Accuracy (Acc), Sensitivity (Se or S+), Positive Predictive Value (PPV or P+) and their harmonic mean F1 defined as follows:

9 $${\rm{PI}}[ \% ]=\frac{{\rm{N}}-{\rm{FP}}-{\rm{FN}}}{{\rm{N}}}\cdot 100$$

10 $${\rm{Acc}}[ \% ]=\frac{{\rm{TP}}}{{\rm{TP}}+{\rm{FP}}+{\rm{FN}}}\cdot 100$$

11 $${\rm{Se}}[ \% ]=\frac{{\rm{TP}}}{{\rm{TP}}+{\rm{FN}}}\cdot 100$$

12 $${\rm{PPV}}[ \% ]=\frac{{\rm{TP}}}{{\rm{TP}}+{\rm{FP}}}\cdot 100$$

13 $${\rm{F}}1[ \% ]=2\cdot \frac{{\rm{PPV}}\cdot {\rm{Se}}}{{\rm{PPV}}+{\rm{Se}}}=2\cdot \frac{{\rm{TP}}}{2{\rm{TP}}+{\rm{FN}}+{\rm{FP}}}\cdot 100$$

where: N – is the total number of the reference FQRS complexes,

TP – is the number of true positive detections (correctly identified),

FP – is the number of false positive detections (extra detected QRS complexes),

FN – is the number of false negative detections (missed QRS complexes).

However, in addition to analyzing data loss in an FHR signal, it is also necessary to assess the accuracy of FQRS complex detection, i.e. the accuracy of instantaneous FHR value measurement^7,25,29^. To assess the accuracy of RR cycles measurement, descriptive statistics of the differences ΔRR between the corresponding RR intervals were calculated by a given detection algorithm. Next, their reference values were determined. It seems to be of key importance to determine a mean absolute measurement error |ΔRR|, defined as the mean of absolute values of differences expressed in both milliseconds and in beats per minute. The value of this error estimates the usefulness of the FHR signal for assessing the clinically important fetal heart rate variability from beat to beat. The last step of the research should be the evaluation whether an FECG morphology analysis (assessment of the amplitude-time relationship between particular waves) is feasible as this provides completely new diagnostic information helpful for verifying the true fetal distress in cases of a recognized suspected or abnormal FHR signal^33,54,57,58^.

What makes it difficult to evaluate the effectiveness of various methods of the indirect FECG signal analysis is a limited number of publicly available testing signals of sufficient quality and duration with reference information provided^18^. For example, the available Daisy database (DDB)^59^ comprises only single records registered in a patient in just 10 seconds, with a sampling frequency of only 250 Hz. The record consists of five abdominal and three thoracic leads and does not contain any expert annotations. The non-invasive fetal electrocardiogram database (NIFECGDB)^60^, available on *PhysioNet*, is larger and contains a series of 55 multichannel abdominal recordings (sampled with 1 kHz), acquired between the 21st and 40th week of pregnancy. Each record has three or four abdominal and two thoracic signals, but only annotations specifying the location of maternal R-waves are available. The Fetal ECG Synthetic Database (FECGSYNDB)^61^ includes 1,750 five-minute simulations of 32 abdominal and two thoracic channels (in total 145.8 hours of data). The simulated data utilized the *fecgsyn simulator*^62^, which comprised seven varying physiological events, for ten different maternal and fetal heart dipole arrangements, at five noise levels. It should be noted that artificially modelled signals, even if the modelled parameters are changed in large ranges, will not fully reflect the nature of real abdominal signals, especially in the case of long-term pregnancy signals^10,14,18,42,43^.

The purpose of this publication is to provide an open access to the bioelectrical fetal heart activity signal datasets^52^ recorded both during pregnancy (B1 dataset) and labour (B2 dataset) with reference heartbeats annotations. An important element of the research material is the reference time markers, verified by clinical experts, indicating the locations of maternal and fetal QRS complexes in signals. Such material seems to be necessary in the process of effective design, optimization or validation of various new methods of suppressing interferences in the abdominal signals, as well as methods of extraction and analysis of the fetal electrocardiogram that has been recorded indirectly.

As part of the work that our team has been conducting in this area for several years now, some of the currently presented data have already been used in our studies. Our initial investigations focused mainly on the methods to suppress the dominant maternal electrocardiogram in abdominal signals. The available literature contains various methods for MECG signal suppression. The methods are often referred to as FECG signal extraction methods because they were not only used to suppress the MECG, but also to reduce the influence of other interferences^12,25,63–66^. In general, all methods for the MECG signal suppression can be divided into three main groups which deploy adaptive filtering, spatial filtering and techniques for subtracting the MECG pattern, as applicable. Different combinations of these methods are also commonly used^19,22,45,48,67–70^. The selection of a given MECG suppression method depends on the electrode configuration. Our research team has made a basic assumption to focus only on methods that do not require additional chest electrodes. The operation of online algorithms, as well as their practical implementation in a small, mobile device for telemedical applications, was also considered to be a very important requirement^5,41^. In the works^35^ we proposed a maternal electrocardiogram suppression method that was based on subtracting the maternal signal template covering the full heart cycle – a PQRST complex. Afterwards, the effectiveness of the method was shown only visually, on a small fragment of a single abdominal signal that had been recorded antenatally. The decisive factor was to precisely determine the fiducial points locating maternal R-waves, as well as to select coefficients for scaling the template during the subtraction process. In a series of experiments described in^40^, we noted a very significant negative impact of episodes with a coincidence of maternal and fetal QRS complexes. This led to the application of preliminary spatial filtering of abdominal signals to create an auxiliary, undisturbed MECG signal. Only the MECG signal was used to determine the locations of maternal R waves and to calculate the weighted coefficients of the maternal template during subtraction. Additionally, to minimize the influence of the sampling frequency of abdominal signals (500 Hz) on the determination of the fiducial points, we proposed an additional subtraction of the first derivative of the maternal QRS complex template. The effectiveness of the original MECG suppression methods with template subtraction was verified in^34^, with the use of dedicated measures and three records from the proposed B1 dataset.

In^37^, the complete abdominal signal processing channel was evaluated to ensure the highest continuity of fetal heart rate signal determination. In addition to the modified MECG suppression method based on the subtraction of the template created using Adaptive Impulse Correlated Filtering (AICF), a new approach with Projective Filtering of Time-Aligned Beats (PFTAB) was also used^20,32,71^. After suppressing the MECG component, the next step was the detection of FQRS complexes. In our research^37^, we examined the detection function based on matched filtering. We considered that way of FQRS feature extraction to be more effective than the classic time-constant bandpass filtering. On the other hand, the normalization of the matched filter output significantly reduced the sensitivity of the detection function to muscle interferences and enabled the selection of the correct peak of the detection function using decision rules based on the cost function minimization. In the above studies, the B1 pregnancy signal dataset, due to the lack of expert verification, was used only to optimize particular parameters for different MECG suppression and FQRS detection algorithms. In turn, the final performance evaluation of the examined methods was verified using three verified records from the presented B2 labour dataset. Among the best methods for processing abdominal signals, FQRS detection efficiency of PI = 81.4% was identified. However, the results were significantly different for each abdominal lead (70.4–99.8%). The detected FQRS complex was considered normal if it was no more distant than ± 40 ms from the reference marker determining the R-wave position. The limit value corresponds to the average of all fetal QRS widths in signals.

The next stage of our research concerned the evaluation of both the effectiveness and accuracy of the fetal R-wave location^36^. As many as 10 signals from the B2 dataset were used in these studies (with reference FHR determined based on the direct FECG). To suppress the MECG, the previously developed method for subtracting the maternal heart cycle template was applied. However, due to the fact that labour is an extreme measurement environment, two problems had to be solved: the prediction of the width of the maternal template for a particular lead and the construction of an additional template in cases of maternal heart arrhythmias. For the detection of FQRS complexes, a modified P&T algorithm was used, popular in adult electrocardiography^51,72,73^, where the detection function was based on a digital filter cascade with the frequency response magnitude adjusted to the spectrum of the FQRS in the analyzed signals. The detection threshold and additional validation criteria for periodicity prediction were selected adaptively. The obtained results confirmed the very high detection efficiency of FQRS complexes equal to Acc = 98% (Se = 98.97%, PPV = 98.99%, PI = 97.97%) and the accuracy of instantaneous FHR measuring – the mean absolute error of the measurement was equal to 2.14 ms (0.63 bpm). The obtained values were considered sufficient to analyze clinically relevant beat-to-beat FHR variability.

As a result of the work^36^, five selected records from the labour dataset were made available to the scientific community via the *PhysioNe*t portal^53^ as *The abdominal and direct fetal electrocardiogram database* (ADFECGDB). Additionally, in order to unify the sampling frequency, the abdominal signals were resampled to 1 kHz. In 2013, those signals were part of the research material in the “Noninvasive Fetal ECG: the PhysioNet/Computing in Cardiology Challenge” to develop effective methods for analyzing the abdominal fetal electrocardiogram. In total, over 50 different papers were submitted as part of the competition, mostly presenting already known methods for the abdominal FECG signal analysis^19,25,74^. The above investigations constitute a reliable validation of the data provided by our team. The technical quality of the data was thoroughly checked and confirmed during the competition, and it was never questioned by its participants in their subsequent publications^48,49,63,65,67,75–77^.

Our last work concerned a comprehensive evaluation of non-adaptive FECG extraction methods based on the model of blind source separation by means of the Principal Component Analysis (PCA) and Independent Component Analysis (ICA)^33^. A comparative study was carried out using all records from the B2 dataset. For the first time, the proposed set of measures was used to quantitatively assess the diversity of the collected research material. The measures enabled the analysis of amplitude relationships of maternal and fetal signals, their relationship to interferences, as well as the assessment of the content of clinically important fetal heart rate patterns. The results obtained in the study confirmed the high usability of non-adaptive methods for fetal electrocardiogram extraction in terms of determining the fetal heart rate from abdominal signals. With the PCA method, the FQRS detection efficiency for the labour dataset (B2) was F1 = 98.56% (Se = 98.27%, PPV = 98.86%, Acc = 97.17%), while for ICA it amounted to F1 = 98.55% (Se = 98.23%, PPV = 98.87%, Acc = 97.13%).

To sum up, the abdominal fetal electrocardiography is currently at a stage of intensive development, and in the near future it may replace, to a large extent, the popular Doppler ultrasound method while ensuring that the accuracy of the fetal heart rate measurement will be similar to that of direct electrocardiography. Of great importance is also the economic aspect; the cost of recording bioelectrical signals is definitely lower. Moreover, it is a more convenient solution for telemedical home care, which is becoming increasingly popular in high risk pregnancy monitoring. We truly hope that the research material presented in detail in this article will translate into the international scientific community’s increased interest in the topic of indirect fetal electrocardiography. The practical use of abdominal fetal electrocardiography in fetal condition monitoring, both in terms of the classic approach based on the analysis of heart rate variability and in terms of an additional analysis of changes in electrocardiogram morphology, is an extremely interesting scientific and research aspect to be studied as a possibility.

## Online-only Tables

**Online-only Table 1:** The values of SNR indices describing diversity of particular records from the B1_Pregnancy_dataset.

Record	Abdominal channel	WM [dB]	WF [dB]	WMF [dB]	WEM	WEF	PN	PM	PF
B1_Pregnancy_01	A1	18.9	5.5	13.5	0.04	0.62	18.7	1458.8	65.8
	A2	17.1	7.0	10.1	0.05	0.34	6.3	323.8	31.5
	A3	10.3	4.0	6.3	0.09	0.31	9.1	98.2	23.2
	A4	19.5	4.9	14.6	0.04	0.55	9.0	798.8	27.5
B1_Pregnancy_02	A1	14.5	3.8	10.6	0.06	0.53	11.6	323.8	28.0
	A2	14.3	4.6	9.7	0.08	0.35	8.9	239.4	25.7
	A3	13.0	4.1	8.8	0.10	0.36	14.0	276.8	36.1
	A4	14.8	3.8	11.0	0.07	0.69	15.9	484.4	38.3
B1_Pregnancy_03	A1	13.8	5.3	8.5	0.07	0.44	8.1	192.3	27.3
	A2	14.8	5.5	9.3	0.07	0.37	2.7	80.9	9.6
	A3	18.9	6.3	12.6	0.06	0.23	4.1	320.7	17.7
	A4	12.4	3.0	9.4	0.14	0.56	7.4	128.7	14.6
B1_Pregnancy_04	A1	12.5	4.8	7.8	0.10	0.49	9.4	167.7	28.1
	A2	13.8	9.0	4.8	0.09	0.23	4.0	97.1	31.9
	A3	17.0	8.7	8.3	0.06	0.20	5.7	281.8	42.0
	A4	6.8	−6.6	13.3	0.30	1.13	35.1	167.0	7.7
B1_Pregnancy_05	A1	19.8	6.8	13.0	0.03	0.25	6.2	595.2	30.0
	A2	18.2	8.9	9.3	0.04	0.18	4.2	271.5	32.0
	A3	12.2	10.6	1.6	0.13	0.09	7.1	117.3	81.6
	A4	19.7	6.2	13.6	0.04	0.20	14.7	1391.6	60.8
B1_Pregnancy_06	A1	11.2	4.2	7.0	0.14	0.43	3.9	52.1	10.5
	A2	11.2	4.9	6.3	0.11	0.18	2.1	27.7	6.5
	A3	15.6	5.0	10.6	0.09	0.17	5.7	206.9	17.9
	A4	13.6	3.5	10.1	0.10	0.43	6.4	146.4	14.4
B1_Pregnancy_07	A1	13.9	2.1	11.8	0.05	0.42	10.1	247.9	16.3
	A2	10.4	0.8	9.7	0.09	0.26	7.6	83.2	9.0
	A3	13.7	3.8	9.9	0.08	0.24	5.4	124.9	12.9
	A4	8.3	−3.6	11.9	0.22	3.32	45.9	310.2	20.1
B1_Pregnancy_08	A1	10.3	−1.9	12.2	0.07	0.41	18.4	196.9	11.9
	A2	7.1	−0.5	7.7	0.10	0.39	7.7	39.9	6.8
	A3	12.3	−2.0	14.3	0.13	0.29	8.3	140.2	5.3
	A4	13.9	−1.8	15.7	0.12	0.57	13.5	331.7	8.9
B1_Pregnancy_09	A1	18.6	0.1	18.5	0.03	1.52	14.7	1068.0	15.0
	A2	17.4	1.7	15.8	0.04	0.80	6.8	373.4	9.9
	A3	14.9	3.0	11.8	0.06	0.41	7.3	221.9	14.6
	A4	16.9	−7.7	24.6	0.06	0.43	16.0	783.7	2.7
B1_Pregnancy_10	A1	13.0	4.0	9.0	0.04	0.89	12.4	249.4	31.3
	A2	13.7	4.1	9.6	0.09	0.70	5.5	130.1	14.2
	A3	18.1	7.3	10.8	0.07	0.21	4.8	306.5	25.7
	A4	14.8	4.8	10.0	0.06	0.58	22.2	676.0	67.1

**Online-only Table 2:** The values of SNR indices describing diversity of particular records from the B2_Labour_dataset.

Record	Abdominal channel	WM [dB]	WF [dB]	WMF [dB]	WEM	WEF	PN	PM	PF
B2_Labour_01	A1	15.5	7.4	8.1	0.10	0.30	9.5	337.1	52.7
	A2	3.2	3.8	−0.6	0.34	0.24	23.9	50.1	57.7
	A3	9.7	5.8	3.9	0.21	0.18	10.6	99.7	40.3
	A4	14.0	9.9	4.1	0.14	0.11	11.7	291.0	114.1
B2_Labour_02	A1	16.1	8.8	7.4	0.09	0.17	33.3	1373.0	250.8
	A2	14.8	7.4	7.4	0.09	0.20	26.5	800.4	144.1
	A3	3.8	−1.8	5.6	0.16	0.40	25.1	60.1	16.5
	A4	15.7	5.5	10.2	0.08	0.24	18.9	699.2	67.4
B2_Labour_03	A1	9.2	1.1	8.1	0.35	0.50	19.4	161.7	25.1
	A2	6.0	−2.1	8.1	0.32	0.50	30.2	119.1	18.5
	A3	3.7	−8.9	12.6	0.34	0.70	15.5	36.3	2.0
	A4	12.2	−1.1	13.3	0.18	0.45	17.6	295.4	13.8
B2_Labour_04	A1	17.4	6.9	10.4	0.10	0.17	29.1	1588.1	144.1
	A2	15.3	5.5	9.8	0.12	0.21	25.9	880.1	92.5
	A3	−0.8	−6.5	5.7	0.21	0.59	95.2	79.0	21.4
	A4	14.8	3.1	11.7	0.08	0.28	18.8	565.6	37.9
B2_Labour_05	A1	15.0	7.4	7.6	0.18	0.19	10.1	316.9	55.4
	A2	1.9	2.4	−0.6	0.52	0.31	39.4	60.9	69.2
	A3	8.2	3.9	4.3	0.23	0.19	16.5	108.2	40.6
	A4	13.7	10.0	3.7	0.18	0.13	13.0	301.2	129.7
B2_Labour_06	A1	17.8	9.4	8.3	0.08	0.11	21.8	1301.0	190.6
	A2	15.9	8.2	7.7	0.09	0.14	19.5	763.3	130.1
	A3	0.9	−9.6	10.5	0.15	0.33	49.1	60.9	5.4
	A4	16.4	6.6	9.9	0.06	0.18	14.0	615.0	63.2
B2_Labour_07	A1	7.9	−3.4	11.3	0.25	1.71	17.4	107.2	8.0
	A2	12.2	2.8	9.4	0.29	0.33	23.5	395.2	45.2
	A3	10.4	0.0	10.5	0.30	0.31	14.5	160.1	14.4
	A4	15.2	2.6	12.6	0.25	0.31	30.4	1007.8	55.6
B2_Labour_08	A1	14.8	7.2	7.6	0.07	0.24	12.1	367.8	63.5
	A2	3.4	2.7	0.8	0.35	0.23	36.5	80.1	67.4
	A3	10.9	8.0	3.0	0.19	0.14	8.4	104.2	52.4
	A4	13.1	10.2	2.9	0.11	0.10	13.8	278.9	144.6
B2_Labour_09	A1	16.3	7.5	8.8	0.11	0.18	34.7	1467.6	194.6
	A2	14.4	6.4	7.9	0.12	0.19	31.7	863.3	139.4
	A3	0.2	−3.6	3.8	0.28	1.18	129.5	135.2	56.5
	A4	14.9	5.4	9.5	0.08	0.21	19.1	595.1	66.0
B2_Labour_10	A1	7.1	−5.5	12.6	0.18	1.05	24.6	127.1	7.0
	A2	12.4	2.8	9.6	0.09	0.32	25.8	445.6	49.1
	A3	10.9	1.4	9.5	0.12	0.26	13.6	168.1	18.7
	A4	15.8	4.2	11.6	0.07	0.30	31.0	1179.2	82.0
B2_Labour_11	A1	14.2	7.1	7.2	0.13	0.18	14.1	374.0	71.5
	A2	−3.9	0.7	−4.5	0.38	0.20	68.5	28.2	80.1
	A3	10.4	7.7	2.7	0.20	0.14	9.3	101.6	54.6
	A4	13.0	11.1	1.9	0.14	0.10	12.9	257.3	167.1
B2_Labour_12	A1	11.1	3.5	7.6	0.19	0.36	28.3	364.3	63.4
	A2	−3.5	−1.9	−1.5	0.68	0.45	116.1	52.4	74.1
	A3	7.2	4.8	2.4	0.32	0.19	17.2	90.0	51.9
	A4	11.1	8.8	2.2	0.27	0.13	18.2	232.8	139.2

## References

[1] Abdulhay E (2014). Non-Invasive Fetal Heart Rate Monitoring Techniques: Review Article. Biomedical Science and Engineering..

[2] Wrobel J, Horoba K, Pander T, Jezewski J, Czabanski R (2013). Improving the fetal heart rate signal interpretation by application of myriad filtering. Biocybern. Biomed. Eng..

[3] Jezewski, J., Wrobel, J. Fetal monitoring with automated analysis of cardiotocogram: the KOMPOR System. Proc. 15th Ann. Int. Conf. IEEE Engineering in Medicine and Biology Society. San Diego, California USA, 28–31 October 1993, 638–639 (1993).

[4] Jezewski J, Kupka T, Horoba K (2008). Extraction of fetal heart rate signal as time event series from evenly sampled data acquired using Doppler ultrasound technique. IEEE Trans. Biomed. Eng..

[5] Jezewski J (2016). Selected design issues of the medical cyber-physical system for telemonitoring pregnancy at home. Microprocess. Microsy..

[6] Kupka, T., Jezewski, J., Matonia, A. & Horoba, K. Timing events in Doppler ultrasound signal of fetal heart activity, Proc. 26th Ann. Int. Conf. IEEE Engineering in Medicine and Biology Society. San Francisco, California USA, 1–5 September 2004, 337–340 (2004). 10.1109/IEMBS.2004.1403161 17271679

[7] Signorini M, Magenes G, Cerutti S, Arduini D (2003). Linear and nonlinear parameters for the analysis of fetal heart rate signal from cardiotocographic recordings. IEEE Trans. Biomed. Eng..

[8] Wrobel J (2015). Medical cyber-physical system for home telecare of high-risk pregnancy – design challenges and requirements. J. Med. Imag. Health. In..

[9] Jongsma HW, van Oosterom A, Murray HG, van Geijn HP (1986). Introduction to fetal electrocardiography. J. Perinat. Med..

[10] Kahankova, R. *et al*. A review of signal processing techniques for non-invasive fetal electrocardiography. *IEEE Rev. Biomed. Eng*. **13**, 51–73 (2019). 10.1109/RBME.2019.2938061 31478873

[11] Hon E (1963). Instrumentation of fetal heart rate and fetal electrocardiography. II. A vaginal electrode. Am. J. Obstet. Gynecol..

[12] Agostinelli A (2015). Noninvasive Fetal Electrocardiography: An Overview of the Signal Electrophysiological Meaning, Recording Procedures, and Processing Techniques. Ann. Noninvas. Electro..

[13] Martinek R (2017). Non-invasive fetal monitoring: a maternal surface ECG electrode placement-based novel approach for optimization of adaptive filter control parameters using the LMS and RLS algorithms. Sensors..

[14] Karvounis E (2010). A non-invasive methodology for fetal monitoring during pregnancy. Methods Inf. Med..

[15] Kahankova R (2017). The influence of gestation age on the performance of adaptive algorithms used for fetal ECG signal extraction. Adv. Elect. Electr. Eng..

[16] Joseph, J., Gini, J. R. & Ramachandran, K. I. Removal of BW and respiration noise in abdECG for fECG extraction. Proc. Int. Symp. on Signal Processing and Intelligent Recognition Systems (SIRS’2017). Thampi, S. M. *et al*., Eds., Advances in Signal Processing and Intelligent Recognition Systems, Manipal, India, 13–16 September 2017, Springer International Publishing AG 2018, 678, 3–14 (2017).

[17] Fotiadou E, van Laar JOEH, Oei SG, Vullings R (2018). Enhancement of low-quality fetal electrocardiogram based on time-sequenced adaptive filtering. Med. Biol. Eng. Comput..

[18] Behar J, Andreotti F, Zaunseder S, Oster J, Clifford GD (2016). A practical guide to non-invasive foetal electrocardiogram extraction and analysis. Physiol. Meas..

[19] Clifford G, Silva I, Behar J, Moody G (2014). Non-invasive fetal ECG analysis. Physiol. Meas..

[20] Kotas M (2008). Combined application of independent component analysis and projective filtering to fetal ECG extraction. Biocybern. Biomed. Eng..

[21] Martinek R. *et al*. Adaptive signal processing techniques for extracting abdominal fetal electrocardiogram, Proc. 10th Int. Symp. on Communication Systems Networks and Digital Signal Processing, (CSNDSP). Prague, Czech Republic, 20–22 July 2016, (2016).

[22] Sameni R, Clifford G (2010). A review of fetal ECG signal processing issues and promising directions. Open Pacing Electrophysiol. Ther. J..

[23] Mousavian I, Shamsollahi MB, Fatemizadeh E (2019). Noninvasive fetal ECG extraction using doubly constrained block-term decomposition. Math. Biosci. Eng..

[24] Castillo E (2018). A clustering-based method for single-channel fetal heart rate monitoring. PLoS ONE.

[25] Behar J, Oster J, Clifford GD (2013). Non-invasive FECG extraction from a set of abdominal sensors. Comput. Cardiol..

[26] Crowe J, Woolfson M, Hayes-Gill B, Peasgood W (1996). Antenatal Assessment using the FECG obtained via Abdominal Electrodes. J. Perinat. Med..

[27] Karvounis E, Tsipouras M, Fotiadis D, Naka K (2007). An automated methodology for fetal heart rate extraction from the abdominal electrocardiogram. IEEE Trans. Inf. Technol. Biomed..

[28] Gacek A, Matonia A, Jezewski J, Kupka T (2007). Recognition of early symptoms of fetal distress with on-line analysis of bioelectrical signals from maternal abdomen. Biocybern. Biomed. Eng..

[29] Jezewski J, Wrobel J, Horoba K (2006). Comparison of Doppler ultrasound and direct electrocardiography acquisition techniques for quantification of fetal heart variability. IEEE Trans. Biomed. Eng..

[30] Jezewski M, Czabanski R, Wrobel J, Horoba K (2010). Analysis of extracted cardiotocographic signal features to improve automated prediction of fetal outcome. Biocybern. Biomed. Eng..

[31] Jezewski M, Czabanski R, Horoba K, Leski J (2016). Clustering with pairs of prototypes to support automated assessment of the fetal state. Appl. Artif. Intell..

[32] Kotas M, Jezewski J, Horoba K, Matonia A (2011). Application of spatio-temporal filtering to fetal electrocardiogram enhancement. Comput. Meth. Prog. Bio..

[33] Martinek R (2018). Comparative effectiveness of ICA and PCA in extraction of fetal ECG from abdominal signals: towards non-invasive fetal monitoring. Front. Physiol..

[34] Matonia, A., Jezewski, J., Horoba, K., Gacek, A. & Labaj, P. The maternal ECG suppression algorithm for efficient extraction of the fetal ECG from abdominal signal. Proc. 28th Ann. Int. Conf. of the IEEE Engineering in Medicine and Biology Society, New York, New York USA, 30 August–3 September 2006, 3106–3109 (2006). 10.1109/IEMBS.2006.260221 17945757

[35] Matonia, A., Jezewski, J., Kupka, T., Horoba, K. & Wrobel, J. Algorithm for recognition and suppression of interfering maternal electrocardiography. Proc. 3rd Int. Conf. on Computer Recognition Systems (KOSYR’2003), Milkow, Poland, 26–29 May 2003, 55–61. ISBN 83-911675-7-7 (2003).

[36] Jezewski J, Matonia A, Kupka T, Roj D, Czabanski R (2012). Determination of the fetal heart rate from abdominal signals: evaluation of beat-to-beat accuracy in relation to the direct fetal electrocardiogram. Biomed. Eng..

[37] Kotas M, Jezewski J, Matonia A, Kupka T (2010). Towards noise immune detection of fetal QRS complexes. Comput. Meth. Prog. Bio..

[38] Matonia, A., Kupka, T., Jezewski, J. & Horoba, K. Evaluation of the QRS detection algorithms in relation to fetal heart rate estimation. Proc. 3rd European Medical & Biological Engineering Conference – IFMBE European Conference on Biomedical Engineering (EMBEC’05). 20-25 November 2005, Prague, Czech Republik, 11 (2005).

[39] Taralunga, D. D., Ungureanu, G. M., Gussi, I., Strungaru, R. & Wolf, W. Fetal ECG extraction from abdominal signals: a review on suppression of fundamental power line interference component and its harmonics. *Comput. Math. Method. M*. Article ID 239060, 15 pages (2014). 10.1155/2014/239060 PMC3934549 24660020

[40] Matonia A, Jezewski J, Kupka T, Horoba K, Wrobel J (2006). The influence of coincidence of fetal and maternal QRS complexes on fetal heart rate reliability. Med. Biol. Eng. Comput..

[41] Wrobel J (2015). Pregnancy telemonitoring with smart control of algorithms for signal analysis. J. Med. Imag. Health In..

[42] Graatsma E, Jacod B, van Egmond L, Mulder E, Visser G (2009). Fetal electrocardiography: feasibility of long-term fetal heart rate recordings. Br. J. Obstet. Gynaecol..

[43] Pieri J (2001). Compact long-term recorder for the transabdominal foetal and maternal electrocardiogram. Med. Biol. Eng. Comput..

[44] Guerrero-Martinez J, Martinez-Sober M, Bataller-Mompean M, Magdaleno-Benedito J (2006). New algorithm for fetal QRS detection in surface abdominal records. Comput. Cardiol..

[45] Jaros R, Martinek R, Kahankova R, Koziorek J (2019). Novel hybrid extraction systems for fetal heart rate variability monitoring based on non-invasive fetal electrocardiogram. IEEE Access..

[46] Marchon, N., Naik, G. & Pai K. R. Linear phase sharp transition BPF to detect noninvasive maternal and fetal heart rate. *J. Healthc. Eng*. Article ID 5485728, 14 pages, (2018). 10.1155/2018/5485728 PMC5896252 29796231

[47] De Haan J (1971). Quantitative evaluation of fetal heart rate patterns I. Processing methods. Europ. J. Obstet. Gynec..

[48] Yan H (2014). Invariant heart beat span versus variant heart beat intervals and its application to fetal ECG extraction. Biomed. Eng. Online..

[49] Almeida R, Goncalves H, Bernardes J, Rocha AP (2014). Fetal QRS detection and heart rate estimation: a wavelet-based approach. Physiol. Meas..

[50] Azavedo S, Longini R (1980). Abdominal-lead fetal electrocardiographic R-wave enhancement for heart rate determination. IEEE Trans. Biomed. Eng..

[51] Agostinelli A (2017). Noninvasive fetal electrocardiography Part I: Pan-Tompkins’ algorithm adaptation to fetal R-peak identification. The Open Biomedical Engineering Journal.

[52] Matonia A (2020). figshare.

[53] Matonia A (2012). PhysioNet.

[54] Jezewski, J. *et al*. A new approach to cardiotocographic fetal monitoring based on analysis of bioelectrical signals. 25th Int. Conf. of IEEE Engineering in Medicine and Biology Society, Cancum, Mexico, 17–21 September 2003, 3145–3149 (2003).

[55] Horoba K (2016). Early predicting a risk of preterm labour by analysis of antepartum electrohysterograhic signals. Biocybern. Biomed. Eng..

[56] Agostinelli A (2017). Noninvasive fetal electrocardiography Part II: Segmented-beat modulation method for signal denoising. The Open Biomedical Engineering Journal.

[57] Fuchs T (2014). Values of T/QRS ratios measured during normal and post-term pregnancies. J. Perinat. Med..

[58] Jaros R, Martinek R, Kahankova R (2018). Non-adaptive methods for fetal ECG signal processing: a review and appraisal. Sensors..

[59] De Moor B, De Gersem P, De Schutter B, Favoreel W (1997). DAISY: A database for identification of systems. Journal A..

[60] Goldberger AL (2000). PhysioBank, PhysioToolkit, and PhysioNet: components of a new research resource for complex physiologic signals. Circulation..

[61] Andreotti F, Behar J, Zaunseder S, Oster J, Clifford GD (2016). An open-source framework for stress-testing non-invasive foetal ECG extraction algorithms. Physiol. Meas..

[62] Behar J (2014). An ECG model for simulating maternal-foetal activity mixtures on abdominal ECG recordings. Physiol. Meas..

[63] Andreotti F (2014). Robust fetal ECG extraction and detection from abdominal leads. Physiol. Meas..

[64] Christov I, Simova I, Abacherli R (2014). Extraction of the fetal ECG in noninvasive recordings by signal decompositions. Physiol. Meas..

[65] Da Poian G, Bernardini R, Rinaldo R (2016). Separation and Analysis of Fetal-ECG Signals From Compressed Sensed Abdominal ECG Recordings. IEEE Trans. Biomed. Eng..

[66] Khamene A, Negahdaripour S (2000). A new method for the extraction of fetal ECG from the composite abdominal signal. IEEE Trans. Biomed. Eng..

[67] Behar J, Oster J, Clifford GD (2014). Combining and benchmarking methods of foetal ECG extraction without maternal or scalp electrode data. Physiol. Meas..

[68] Vullings R (2010). A robust physiology-based source separation method for QRS detection in low amplitude fetal ECG recordings. Physiol. Meas..

[69] Li, C., Fang, B., Li, H. & Wang, P. A novel method of FECG extraction combined self-correlation analysis with ICA. Proc. 8th IEEE Int. Conf. on Communication Software and Networks (ICCSN). Beijing, China 4–6 June 2016, 107–111 (2016).

[70] Liu G, Luan Y (2015). An adaptive integrated algorithm for noninvasive fetal ECG separation and noise reduction based on ICA-EEMD-WS. Med. Biol. Eng. Comput..

[71] Kotas M (2007). Projective Filtering for Fetal ECG extraction. Bull. Pol. Acad. Sci.Te..

[72] Friesen G (1990). A comparison of the noise sensitivity of nine QRS detection algorithms. IEEE Trans. Biomed. Eng..

[73] Pan J, Tompkins W (1985). A real-time QRS detection algorithm. IEEE Trans. Biomed. Eng..

[74] Silva I (2013). Noninvasive Fetal ECG: the PhysioNet/Computing in Cardiology Challenge 2013. Comput. Cardiol..

[75] Rodrigues R (2014). Fetal beat detection in abdominal ECG recordings: global and time adaptive approaches. Physiol. Meas..

[76] Lee K, Lee B (2016). Sequential total variation denoising for the extraction of fetal ECG from single-channel maternal abdominal ECG. Sensors..

[77] John RG, Ramachandran KI (2019). Extraction of foetal ECG from abdominal ECG by nonlinear transformation and estimations. Comput. Meth. Prog. Bio..

